# Supplementary material for: Maternal Mental Health Symptom Profiles and Infant Sleep: A Cross-Sectional Survey
Source: Diagnostics (Basel). 2022 Jul 4;12(7):1625. doi: 10.3390/diagnostics12071625 (PMC9319039; doi:10.3390/diagnostics12071625)
Supplement: Supplementary file 1 [file diagnostics-12-01625-s001.zip › diagnostics-1726980-supplementary.pdf]

diagnostics

MDPI

Supplementary material

(A)

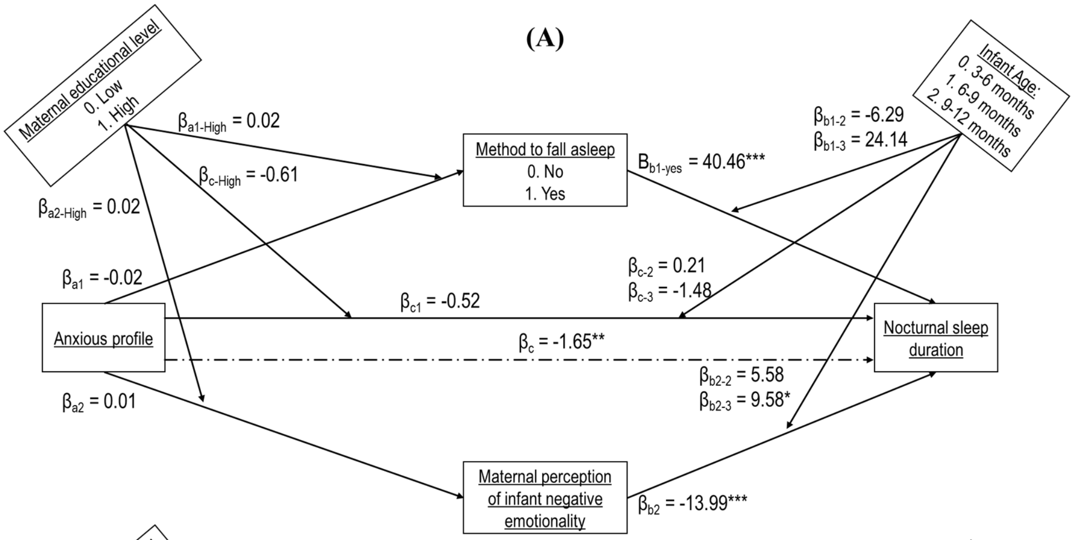

(B)

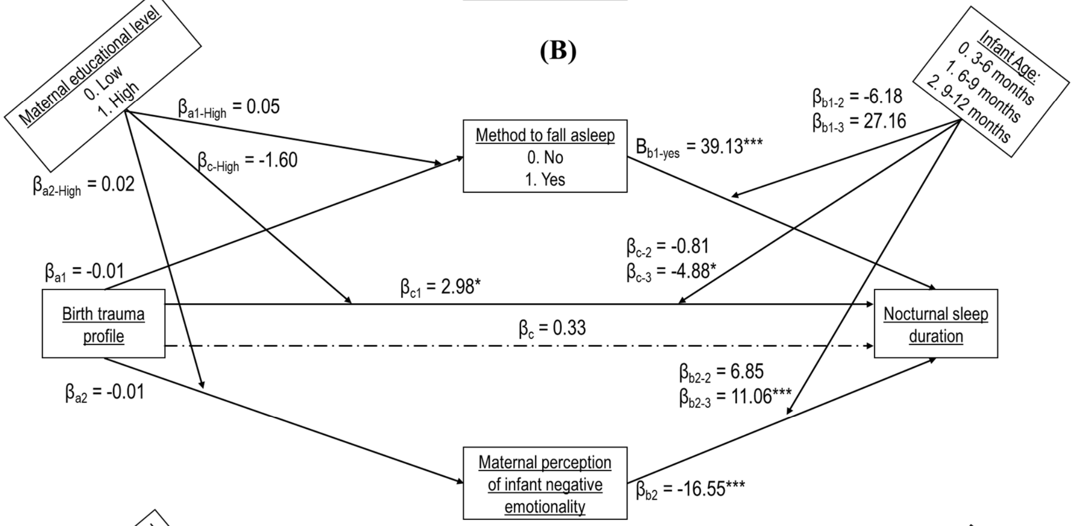

(C)

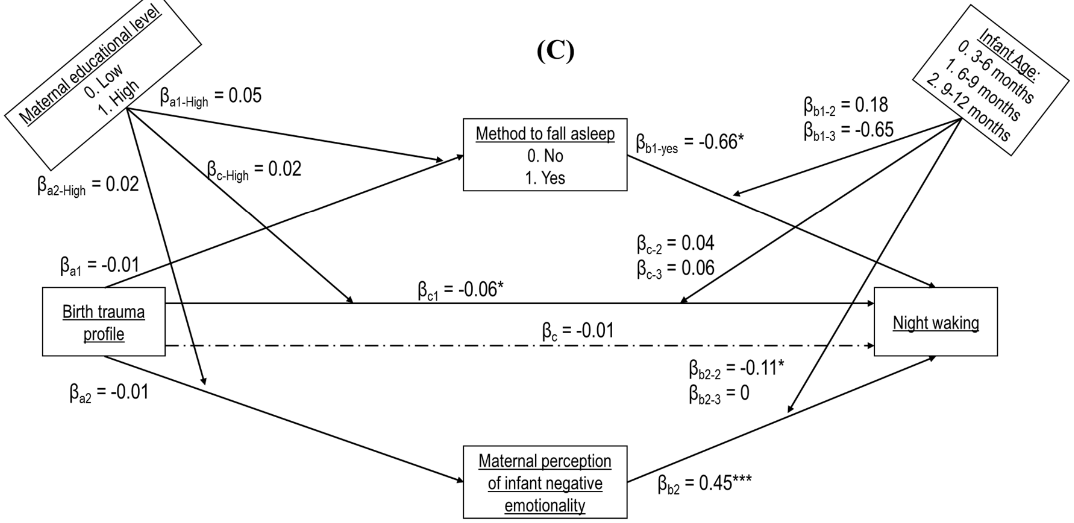

Figure S1. Model (**A**) displays the path model of the influence of the anxious profile on nocturnal sleep duration, including mediators and moderators. Model (**B**) presents the path model of the prediction of nocturnal sleep duration by birth trauma profile, taking into account mediators and moderators. Model (**C**) represents the path model of the influence of the birth trauma profile on night waking, including mediators and moderators. Maternal perception of infant negative emotionality and the method to fall asleep are tested as mediators for all models, as well as maternal educational level and infant age as moderators. Dashed lines show the direct association between the maternal mental health symptom profile and infant sleep without including the other factors in the model. Non standardized beta coefficients are reported. * $p < 0.05$; ** $p < 0.001$; *** $p < 0.001$.

Table S1. Non-significant moderated mediation models investigating relationships between maternal mental health symptom profiles and infant sleep.

Independent variable	Dependent variable	Mediator	Covariate	Moderator	ACME, 95% CI	p	Power Monte Carlo (Sobel)
**Model 3**							
Depressive profile	Nocturnal sleep duration	IBQ-NEG	Method to fall asleep	Infant age: ≥3 months to <6 months	−0.249, [−0.737, 0.239]	0.292	0.48 (0.20)
Depressive profile	Nocturnal sleep duration	IBQ-NEG	Method to fall asleep	Infant age: ≥9 months to <12 months	−0.047, [−0.439, 0.177]	0.768	0.12 (0.07)
**Model 4**							
Anxious profile	Night waking	IBQ-NEG	Method to fall asleep	Infant age: ≥6 months to <9 months	0.003, [−0.005, 0.014]	0.524	0.10 (0.11)
Anxious profile	Night waking	IBQ-NEG	Method to fall asleep	Infant age: ≥9 months to <12 months	0.009, [−0.003, 0.026]	0.236	0.24 (0.21)
**Model 5**							
Anxious profile	Night waking	IBQ-NEG	Method to fall asleep	Low educational level	0.009, [0, 0.021]	0.072	0.42 (0.33)
**Model 6**							
Depressive profile	Night waking	Method to fall asleep	IBQ-NEG	Low educational level	0.005, [0, 0.013]	0.1	0.41 (0.35)
Depressive profile	Night waking	Method to fall asleep	IBQ-NEG	High educational level	0.003 [0, 0.007]	0.08	0.38 (0.36)
**Model 7**							
Depressive profile	Nocturnal sleep duration	Method to fall asleep	IBQ-NEG	Low educational level	−0.226, [−0.772, 0]	0.108	0.23 (0.28)

	Depressive profile	Nocturnal sleep duration	Method to fall asleep	IBQ-NEG	High educational level	−0.242. [−0.548, 0.012]	0.076	0.41 (0.38)
**Model 8**								
	Depressive profile	Nocturnal sleep duration	IBQ-NEG	Method to fall asleep	Low educational level	−0.217, [−0.681, 0.107]	0.324	0.24 (0.17)
	Depressive profile	Nocturnal sleep duration	IBQ-NEG	Method to fall asleep	High educational level	−0.058, [−0.359, 0.14]	0.572	0.10 (0.09)
**Model 9**								
	Birth trauma profile	Night waking	Method to fall asleep	IBQ-NEG	Low educational level	0.007, [−0.004, 0.022]	0.248	0.21 (0.19)
	Birth trauma profile	Night waking	Method to fall asleep	IBQ-NEG	High educational level	−0.009, [−0.019, 0]	0.064	0.50 (0.43)
**Model 10**								
	Birth trauma profile	Night waking	IBQ-NEG	Method to fall asleep	Low educational level	0.001, [−0.012, 0.014]	0.872	0.06 (0.06)
	Birth trauma profile	Night waking	IBQ-NEG	Method to fall asleep	High educational level	0.002, [−0.009, 0.013]	0.732	0.06 (0.06)
**Model 11**								
	Birth trauma profile	Nocturnal sleep duration	Method to fall asleep	IBQ-NEG	Low educational level	−0.361, [−1.263, 0.207]	0.3	0.14 (0.18)
	Birth trauma profile	Nocturnal sleep duration	Method to fall asleep	IBQ-NEG	High educational level	0.668, [−0.012, 1.455]	0.064	0.53 (0.47)
**Model 12**								
	Birth trauma profile	Nocturnal sleep duration	IBQ-NEG	Method to fall asleep	Low educational level	−0.033, [−0.69, 0.275]	0.872	0.02 (0.06)
	Birth trauma profile	Nocturnal sleep duration	IBQ-NEG	Method to fall asleep	High educational level	−0.021, [−0.355, 0.076]	0.78	0.05 (0.06)

**Model 13**							
Anxious profile	Night waking	Method to fall asleep	IBQ-NEG	Low educational level	0.01, [0.001, 0.025]	0.06	0.43 (0.40)
Anxious profile	Night waking	Method to fall asleep	IBQ-NEG	High educational level	−0.001, [−0.007, 0.006]	0.848	0.06 (0.05)
**Model 14**							
Anxious profile	Nocturnal sleep duration	Method to fall asleep	IBQ-NEG	Low educational level	−0.489, [−1.394, −0.015]	0.068	0.30 (0.33)
Anxious profile	Nocturnal sleep duration	Method to fall asleep	IBQ-NEG	High educational level	0.042, [−0.434, 0.582]	0.896	0.05 (0.05)
**Model 15**							
Anxious profile	Nocturnal sleep duration	IBQ-NEG	Method to fall asleep	Low educational level	−0.237, [−1.152, 0.06]	0.284	0.15 (0.18)
Anxious profile	Nocturnal sleep duration	IBQ-NEG	Method to fall asleep	High educational level	−0.08, [−0.334, 0.107]	0.38	0.18 (0.14)
**Model 16**							
Depressive profile	Night waking	Method to fall asleep	IBQ-NEG	Infant age: ≥3 months to <6 months	0.001, [−0.004, 0.006]	0.648	0.07 (0.08)
Depressive profile	Night waking	Method to fall asleep	IBQ-NEG	Infant age: ≥6 months to <9 months	0.004, [0, 0.012]	0.052	0.54 (0.34)
Depressive profile	Night waking	Method to fall asleep	IBQ-NEG	Infant age: ≥9 months to <12 months	0.005, [−0.003, 0.014]	0.292	0.21 (0.18)
**Model 17**							
Depressive profile	Nocturnal sleep duration	Method to fall asleep	IBQ-NEG	Infant age: ≥3 months to <6 months	−0.071, [−0.459, 0.199]	0.596	0.04 (0.08)
Depressive profile	Nocturnal sleep duration	Method to fall asleep	IBQ-NEG	Infant age: ≥6 months to <9 months	−0.177, [−0.609, 0.019]	0.152	0.46 (0.27)

Depressive profile	Nocturnal sleep duration	Method to fall asleep	IBQ-NEG	Infant age: ≥9 months to <12 months	−0.245, 0.164]	[−0.681,	0.24	0.20 (0.18)
**Model 18**								
Birth trauma profile	Night waking	Method to fall asleep	IBQ-NEG	Infant age: ≥3 months to <6 months	−0.001, 0.008]	[−0.015,	0.828	0.04 (0.06)
Birth trauma profile	Night waking	Method to fall asleep	IBQ-NEG	Infant age: ≥6 months to <9 months	−0.003, 0.003]	[−0.015,	0.408	0.13 (0.14)
Birth trauma profile	Night waking	Method to fall asleep	IBQ-NEG	Infant age: ≥9 months to <12 months	−0.001, 0.018]	[−0.023,	0.924	0.05 (0.05)
**Model 19**								
Birth trauma profile	Night waking	IBQ-NEG	Method to fall asleep	Infant age: ≥3 months to <6 months	0.006, 0.021]	[−0.005,	0.376	0.12 (0.13)
Birth trauma profile	Night waking	IBQ-NEG	Method to fall asleep	Infant age: ≥6 months to <9 months	0.003, 0.017]	[−0.006,	0.628	0.10 (0.08)
Birth trauma profile	Night waking	IBQ-NEG	Method to fall asleep	Infant age: ≥9 months to <12 months	−0.009, 0.009]	[−0.046,	0.236	0.15 (0.14)
**Model 20**								
Birth trauma profile	Nocturnal sleep duration	Method to fall asleep	IBQ-NEG	Infant age: ≥3 months to <6 months	0.08, 0.838]	[−0.481,	0.872	0.05 (0.06)
Birth trauma profile	Nocturnal sleep duration	Method to fall asleep	IBQ-NEG	Infant age: ≥6 months to <9 months	0.153, 0.671]	[−0.141,	0.36	0.11 (0.13)
Birth trauma profile	Nocturnal sleep duration	Method to fall asleep	IBQ-NEG	Infant age: ≥9 months to <12 months	0.048, 1.091]	[−0.956,	0.892	0.05 (0.09)
**Model 21**								
Birth trauma profile	Nocturnal sleep duration	IBQ-NEG	Method to fall asleep	Infant age: ≥3 months to <6 months	−0.158, 0.131]	[−0.733,	0.476	0.08 (0.12)
Birth trauma profile	Nocturnal sleep duration	IBQ-NEG	Method to fall asleep	Infant age: ≥6 months to <9 months	−0.155, 0.314]	[−0.968,	0.608	0.08 (0.08)

Birth trauma profile	Nocturnal sleep duration	IBQ-NEG	Method to fall asleep	Infant age: ≥9 months to <12 months	0.12,	[−0.243, 0.599]	0.656	0.06 (0.11)
**Model 22**								
Anxious profile	Night waking	Method to fall asleep	IBQ-NEG	Infant age: ≥3 months to <6 months	0.003,	[−0.005, 0.012]	0.42	0.09 (0.10)
Anxious profile	Night waking	Method to fall asleep	IBQ-NEG	Infant age: ≥6 months to <9 months	−0.003,	[−0.002, 0.012]	0.312	0.15 (0.16)
Anxious profile	Night waking	Method to fall asleep	IBQ-NEG	Infant age: ≥9 months to <12 months	0,	[−0.015, 0.016]	0.94	0.06 (0.05)
**Model 23**								
Anxious profile	Nocturnal sleep duration	Method to fall asleep	IBQ-NEG	Infant age: ≥3 months to <6 months	−0.174,	[−0.946, 0.232]	0.544	0.09 (0.10)
Anxious profile	Nocturnal sleep duration	Method to fall asleep	IBQ-NEG	Infant age: ≥6 months to <9 months	0.151,	[−0.719, 0.114]	0.368	0.13 (0.15)
Anxious profile	Nocturnal sleep duration	Method to fall asleep	IBQ-NEG	Infant age: ≥9 months to <12 months	0.003,	[−0.796, 0.874]	0.924	0.05 (0.05)
**Model 24**								
Anxious profile	Nocturnal sleep duration	IBQ-NEG	Method to fall asleep	Infant age: ≥3 months to <6 months	−0.283,	[−1.052, 0.14]	0.284	0.32 (0.21)
Anxious profile	Nocturnal sleep duration	IBQ-NEG	Method to fall asleep	Infant age: ≥6 months to <9 months	−0.172,	[−0.822, 0.28]	0.504	0.09 (0.11)
Anxious profile	Nocturnal sleep duration	IBQ-NEG	Method to fall asleep	Infant age: ≥9 months to <12 months	−0.081,	[−0.707, 0.081]	0.58	0.05 (0.10)

Note. ACME: Average Causal Mediation Effects; IBQ-NEG = negative emotionality subscale of the Very Short Form of the Infant Behavior Questionnaire-Revised. Post hoc power analyses for testing mediation effect based on 1000 Monte Carlo simulations (Thoemmes, F. MacKinnon, D. & Reiser, M., 2010) and based on Sobel's test (Sobel, 1982) [1,2].

References

1. Sobel, M.E. Asymptotic Confidence Intervals for Indirect Effects in Structural Equation Models. *Sociol. Methodol.* **1982**, *13*, 290–312. https://doi.org/10.2307/270723.
2. Thoemmes, F.; MacKinnon, D.P.; Reiser, M.R. Power Analysis for Complex Mediational Designs Using Monte Carlo Methods. *Struct. Equ. Modeling Multidiscip. J.* **2010**, *17*, 510–534. https://doi.org/10.1080/10705511.2010.489379.
